# Evaluating the Role of Morphological Subtypes in the Classification of Periampullary Adenocarcinomas

**DOI:** 10.3390/cancers17223652

**Published:** 2025-11-14

**Authors:** João Bernardo Sancio, Raul Valério Ponte, Henrique Araújo Lima, Augusto Henrique Marchiodi, Yuiti Pedro Henrique Yamashita, Leonardo do Prado Lima, Priscila Ferreira de Lima e Souza, Eduardo Paulino Junior, Marcelo Dias Sanches, Vivian Resende

**Affiliations:** 1 Department of Surgery, School of Medicine, Federal University of Minas Gerais, Belo Horizonte 30130-100, MG, Brazil; joaosancio@ufmg.br (J.B.S.); lima.henriquea@gmail.com (H.A.L.); augustomarchiodi@ufmg.br (A.H.M.); yuitipedro@ufmg.br (Y.P.H.Y.); drleonardoprado@gmail.com (L.d.P.L.); msanches@mac.com (M.D.S.); 2 Department of General Surgery, Institute Dr. José Frota (IJF), Fortaleza 60025-061, CE, Brazil; rppp@live.com; 3 Department of Anesthesiology, School of Medicine, Federal University of Ceará, Fortaleza 60020-181, CE, Brazil; priscilaferreiramed@gmail.com; 4 Department of Clinical Pathology, School of Medicine, Federal University of Minas Gerais, Belo Horizonte 30130-100, MG, Brazil; edupatol@gmail.com

**Keywords:** periampullary neoplasms, pancreaticoduodenectomy, adenocarcinoma, histological subtypes, lymph nodes, prognosis

## Abstract

**Background**: Morphological subclassification may refine prognosis after curative pancreaticoduodenectomy (PD) for periampullary cancers. **Methods**: We conducted a single-center retrospective cohort including 120 consecutive PDs performed between 2005 and 2022. Tumors were classified as intestinal (INT), pancreatobiliary (PB), or pancreatic ductal adenocarcinoma (PAN). Clinicopathologic variables included T stage, margin status, lymphovascular and perineural invasion, and lymph node ratio (LNR; cutoff 0.154 determined by ROC/Youden). Overall survival (OS) was the primary endpoint and was analyzed using Kaplan–Meier with log-rank tests and multivariable Cox regression. **Results**: INT tumors were associated with earlier T stage, fewer adverse histologic features, and higher R0 resection rates compared with PB and PAN. In multivariable analysis, mortality risk was higher for PB (HR 4.41; 95% CI 1.25–15.53) and PAN (HR 13.96; 95% CI 3.99–48.75) relative to INT. LNR ≥ 0.154 independently predicted worse OS (HR 1.93; 95% CI 1.11–3.35). Mean OS was 108.8 months for INT, 62.0 months for PB, and 22.7 months for PAN (log-rank *p* < 0.001). **Conclusions**: Morphological subtype and LNR are independent prognostic factors after PD for periampullary malignancies. Integrating morphology and nodal burden into risk models may improve postoperative stratification and guide adjuvant therapy.

## Simple Summary

Periampullary adenocarcinomas are uncommon cancers that develop near the junction of the pancreas, bile duct, and small intestine. Although surgery is the main treatment, survival varies widely among patients, even when tumors appear similar. This study analyzed 120 patients who underwent curative surgery at a university hospital to explore whether microscopic tumor appearance could predict patient outcomes. We found that patients whose tumors showed an intestinal pattern lived much longer than those with pancreatobiliary or pancreatic patterns. Tumors with more lymph node involvement also had worse survival. These findings show that microscopic tumor subtype and lymph node burden provide valuable information for predicting prognosis after surgery. Incorporating these factors into clinical practice may help doctors better estimate survival, select adjuvant treatments, and design more precise studies in the future.

## 1. Introduction

Periampullary adenocarcinomas comprise four adjacent anatomic sites: ampulla of Vater, distal common bile duct, duodenum, and pancreatic head ductal adenocarcinoma [1,2]. Surgical resection remains the principal therapeutic option to improve survival and offer a cure, most commonly via pancreaticoduodenectomy in resectable cases [3]. Traditionally, these tumors are staged by anatomic origin according to the American Joint Committee on Cancer (AJCC) [4]. Nonetheless, survival after resection varies substantially even among neoplasms of the same site [5,6], and anatomic proximity can hinder precise identification of the primary tumor, with implications for adjuvant treatment selection [7].

To address heterogeneity beyond anatomic site, Kimura et al. proposed two histomorphologic patterns in ampullary carcinoma—intestinal (INT) and pancreatobiliary (PB) [8]. On routine hematoxylin–eosin, INT tumors resemble gastric/colonic tubular adenocarcinoma with elongated, well-formed glands and basally oriented oval nuclei, whereas PB tumors exhibit papillary projections with delicate fibrovascular cores; immunophenotypically, INT commonly expresses CDX2 and rarely MUC1, while PB shows strong MUC1 with weak/absent CDX2 [6,9]. INT tumors tend to be less infiltrative and have better outcomes than PB, and morphological subclassification is increasingly considered when selecting postoperative systemic therapy, particularly for ampullary disease [8,10].

However, the prognostic value of morphology across periampullary sites remains uncertain. Histomorphologic subclassification has demonstrated prognostic value in other malignancies, such as gastric and colorectal adenocarcinomas, suggesting that similar morphological frameworks may also enhance prognostic evaluation in ampullary and pancreatic ductal adenocarcinoma (PDAC). PDAC’s biological heterogeneity and the lack of robust prognostic markers beyond TNM staging continue to challenge risk stratification and therapeutic decision-making [8,10].

Pancreatic ductal adenocarcinoma exhibits the poorest post-resection outcomes (≈5–20% five-year survival), whereas ampullary adenocarcinoma reaches ≈33–68%; [5,10,11] some studies report no survival difference between pancreatic adenocarcinoma (PAN) and ampullary PB subtype [6]. In this context, we aimed to determine whether morphological subtype (INT, PB, and PAN) is associated with distinct clinicopathologic features and overall survival in a single-center cohort of patients undergoing curative-intent pancreaticoduodenectomy between 2005 and 2022, and to evaluate whether lymph node ratio provides additional prognostic stratification.

## 2. Methods

### 2.1. Study Design and Setting

This was a single-center, longitudinal, retrospective cohort study conducted at a tertiary academic hospital. Data were prospectively collected in institutional protocols and medical records from 2005 to 2022.

All procedures complied with institutional and national research ethics. The study was approved by the Research Ethics Committee (COEP/UFMG), protocol CAAE 23377113100005149; informed consent was waived due to the retrospective design.

### 2.2. Patients and Eligibility

**Figure 1:**
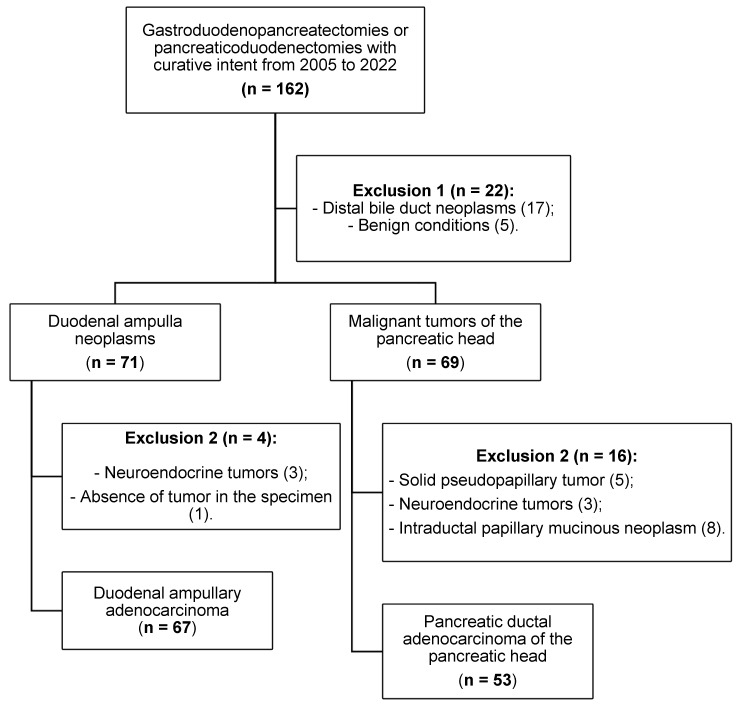
Patient selection and application of inclusion/exclusion criteria.

We screened 162 consecutive patients considered for curative resection and obtained a final cohort of 120 after applying eligibility criteria (Figure 1). Inclusion criteria: ductal adenocarcinoma of the pancreatic head and ampullary adenocarcinoma treated with pancreaticoduodenectomy. Exclusion criteria: distal common bile duct or duodenal primary tumors; benign conditions; non-ductal pancreatic neoplasms (e.g., solid pseudopapillary tumor, neuroendocrine tumors, intraductal papillary mucinous neoplasm); and non-adenocarcinoma papillary tumors. Adjuvant chemotherapy was indicated and managed by the institutional Medical Oncology Service according to prevailing protocols during the study period.

### 2.3. Variables and Definitions

We collected clinical-demographic variables (age, sex, smoking, alcohol use), preoperative risk by ASA physical status classification [12], and postoperative complications graded by Clavien–Dindo (validated Portuguese translation) [13]. Preoperative laboratory data comprised CA 19-9, total and direct bilirubin, alkaline phosphatase, GGT, AST, ALT, albumin, hemoglobin, leukocyte and lymphocyte counts, and platelets. Histopathologic assessment followed AJCC 8th-edition TNM criteria for exocrine pancreatic and ampullary cancers [4], recording tumor grade, lymphovascular invasion (LVI), perineural invasion (PNI), and resection margin status (R0/R1). The lymph node ratio (LNR) was defined as the number of positive lymph nodes divided by the total retrieved and was analyzed categorically using the prespecified study cut-off.

### 2.4. Histopathologic Review and Morphological Classification

**Figure 2:**
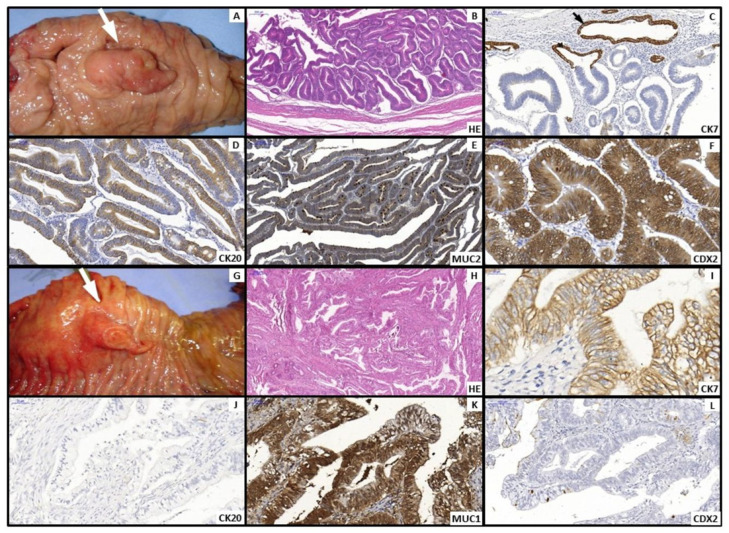
(**A**–**F**). Intestinal type: (**A**) Polypoid Vater’s ampulla tumor (White arrow). (**B**) Microscopy—Hematoxylin and Eosin (H&E) staining: irregular gland-forming adenocarcinoma composed of elongated cells with stratified nuclei and scattered goblet cells. Immunohistochemical profile: CK7 negative, black arrow shows CK7 positive control cells of peribiliary glands (**C**); CK20 (**D**), MUC2 (**E**), and CDX2 (**F**) positive. (**G**–**L**). Pancreatobiliary type: (**G**) Polypoid and eroded tumor of Vater’s ampulla (white arrow). (**H**) Microscopy—Hematoxylin and Eosin (H&E) staining: irregularly infiltrating glands with pale cytoplasm. Immunohistochemical profile: CK7 positive (**I**); CK20 negative (**J**), MUC1 positive (**K**), and CDX2 negative (**L**).

All slides were reviewed by an experienced pathologist at the institutional Pathology Department. Morphological subtype for ampullary tumors was assigned as intestinal (INT) or pancreatobiliary (PB) based primarily on hematoxylin–eosin (H&E) features, complemented by immunohistochemistry (MUC1, MUC2, MUC5AC, CK7, CK20, CDX2) when necessary; from the 33rd case onward, immunohistochemistry was reserved for equivocal H&E classifications (Figure 2). Pancreatic ductal adenocarcinoma (PAN) was analyzed as a distinct group. Staging followed the AJCC 8th edition [4].

### 2.5. Outcomes

The primary outcome was overall survival (OS), measured in months from surgery to death from any cause or last follow-up. For survival analyses, deaths within 30 postoperative days were excluded. OS at 12, 36, and 60 months was also estimated.

### 2.6. Statistical Analysis

Descriptive statistics used means/SD or medians/IQR and frequencies, as appropriate. Group comparisons employed χ^2^ or Fisher’s exact tests (categorical), Kruskal–Wallis and Mann–Whitney (non-parametric continuous), or Student’s *t*-test (parametric). To assess the prognostic performance of LNR, we constructed ROC curves and selected the optimal cutoff by Youden’s index, identifying LNR ≥ 0.154 (sensitivity 51.8%, specificity 85.5%) as the best discriminator for death during follow-up. Survival was estimated by Kaplan–Meier with log-rank tests; covariates with *p* ≤ 0.20 in univariable analyses and/or clinical relevance entered a multivariable Cox proportional hazards model. Two-sided *p* < 0.05 defined statistical significance. Analyses were performed in IBM SPSS^®^ v23 for Mac (Chicago, IL, USA).

## 3. Results

### 3.1. Cohort and Perioperative Outcomes

Of 162 screened cases, 120 patients met eligibility criteria and were included (Figure 1). Baseline clinical characteristics and postoperative outcomes by group are summarized in Table 1. The study cohort comprised 34 intestinal (INT), 33 pancreatobiliary (PB), and 53 pancreatic ductal adenocarcinoma (PAN) cases. The PB subgroup showed a female predominance (66.6%), whereas PAN had more males (62.3%; *p* = 0.019). Smoking and alcohol use were more frequent in PAN than PB (non-smokers: 45.3% vs. 69.7%; *p* = 0.027; alcohol use: 43.4% vs. 15.2%; *p* = 0.007). Most complications were minor (Clavien–Dindo I–II–IIIA); 30-day mortality was low and similar across groups (INT 11.8%, PB 6.1%, PAN 5.7%; *p* = 0.536).

### 3.2. Preoperative Laboratory Profile

Median CA 19-9 was lower in INT versus PAN (20 vs. 267.5 U/mL; *p* < 0.001), while PB was intermediate (29.5 U/mL; overall *p* = 0.001). Total and direct bilirubin were highest in PAN (medians 11.6 and 9.9 mg/dL, respectively) and lowest in INT (0.8 and 0.3 mg/dL; both overall *p* < 0.001). GGT, AST, and ALT were also higher in PAN/PB than INT (e.g., GGT medians 472.5/PB 365.5/INT 147.5 U/L; *p* = 0.022). Albumin, hemoglobin, leukocytes, lymphocytes, and platelets did not differ significantly between groups.

### 3.3. Pathologic Findings

Details are presented in Table 2. INT tumors were predominantly T1 (55.9%), whereas PAN cases were mainly T3–T4 (≥70%; *p* < 0.001). Nodal status differed substantially: N0 was more frequent in INT than PAN (76.5% vs. 30.2%; *p* < 0.001); overall node positivity was highest in PAN (69.8%) versus PB (45.5%) and INT (23.5%). Limited liver metastatic disease was observed intraoperatively (M1: PB 1 case; PAN 5 cases). Margins were more often positive in PAN (35.8%) versus INT (0%) and PB (3%; *p* < 0.001). Lymphovascular and perineural invasion were frequent in PAN (84.9% and 96.2%) and uncommon in INT (~12% each; both *p* < 0.001). Lymph node ratio (LNR) < 0.154 predominated in INT (91.2%) versus PB (69.7%) and PAN (64.2%; overall *p* = 0.018).

### 3.4. Survival Analysis

No statistically significant difference in survival was observed for any of the clinical variables considered. Deaths within 30 postoperative days were excluded from survival analyses. Survival analysis according to the pathological variables studied is presented in Table 3.

**Figure 3:**
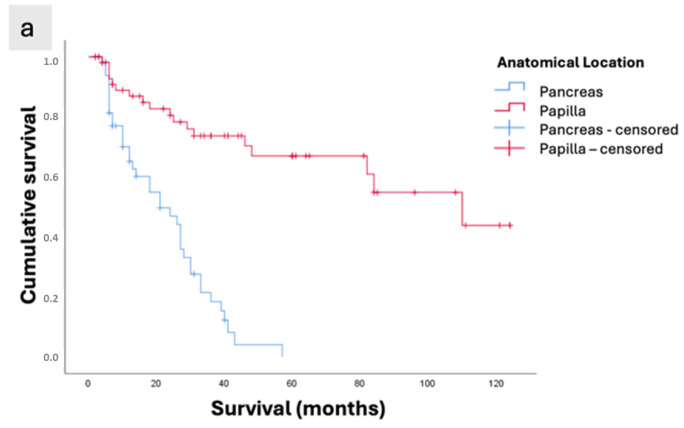

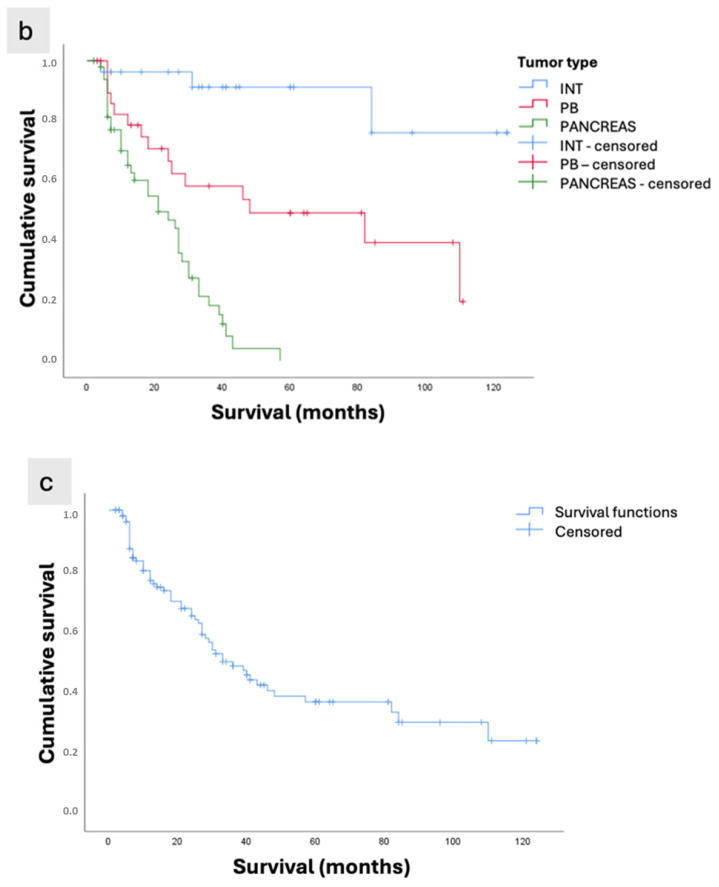
Kaplan–Meier survival curves of patients with periampullary adenocarcinomas who underwent curative-intent surgical resection. (**a**) Survival according to anatomical location (Papilla vs. Pancreas; *p* < 0.001). (**b**) Survival according to morphological subtype (INT: intestinal subtype of ampullary tumors; PB: pancreatobiliary subtype of ampullary tumors; Pancreas: pancreatic ductal adenocarcinoma; *p* < 0.001). (**c**) Overall survival of the study population.

Both the anatomical location (papilla and pancreas) and the morphological classification (intestinal, pancreatobiliary, and pancreatic) were factors that impacted survival according to the univariate analysis (*p* < 0.001). The papilla group had a mean survival of 83.7 months, whereas the pancreas group showed a survival of 22.7 months (Figure 3a).

The analysis according to histopathological phenotype showed that tumors with the best mean survival were of the intestinal subtype (108.7 months), compared with 62 months for the pancreatobiliary subtype and only 22.7 months for pancreatic tumors (*p* < 0.001, Figure 3b).

The mean overall survival was 56.37 ± 5.43 months, with survival rates of 76.9%, 48.8%, and 37% at 12, 36, and 60 months, respectively (Figure 3c).

The intestinal subtype showed the longest survival (108.76 ± 8.04 months), followed by the pancreatobiliary subtype (62.04 ± 8.94 months), while patients with pancreatic ductal adenocarcinoma had the poorest survival (22.70 ± 2.24 months), *p* < 0.001 (Table 4). In the six cases in which metastatic disease was resected, liver metastases were identified intraoperatively and were located in regions favorable to resection. Among these, there was a single case of PB-type ampullary adenocarcinoma, with a postoperative survival of 8 months. In the remaining five cases of pancreatic adenocarcinoma, patient survival times were 41, 18, 7, 6, and 5 months, respectively.

### 3.5. Multivariable Analysis

In Cox regression (Table 5), morphological subtype and LNR remained independent prognostic factors. Compared with INT, the hazard of death was higher for PB (HR 4.41; 95% CI 1.25–15.53; *p* = 0.021) and highest for PAN (HR 13.96; 95% CI 3.99–48.75; *p* < 0.001). LNR ≥ 0.154 independently predicted worse OS (HR 1.93; 95% CI 1.11–3.35; *p* = 0.018).

## 4. Discussion

In this single-center cohort of patients undergoing curative-intent pancreaticoduodenectomy (PD) for periampullary malignancies, morphological subtype emerged as an independent determinant of overall survival (OS): intestinal (INT) tumors had the most favorable outcomes, whereas pancreatobiliary (PB) and pancreatic ductal adenocarcinoma (PAN) displayed progressively worse survival. In multivariable analysis, mortality risk was higher for PB and highest for PAN when both were compared with INT, and lymph node ratio (LNR) ≥ 0.154 independently predicted inferior OS. Together, morphology and LNR refined risk beyond conventional clinicopathologic variables.

These findings support a phenotype-oriented approach to prognostication, complementing anatomic-site staging and addressing the heterogeneity observed within periampullary cancers. Originally described in ampullary carcinoma, the INT versus PB dichotomy is defined by distinct H&E architecture and immunophenotype (e.g., CDX2/MUC1 patterns) and has been linked to clinical behavior [6]. INT tumors in our series presented more favorable pathology (earlier T stage, more frequent N0, lower rates of lymphovascular/perineural invasion, and higher R0 resections), while PB tracked closer to the pancreatic phenotype, and PAN remained the worst-prognosis group—patterns that help explain the graded survival across INT → PB → PAN.

Nodal burden remains central in periampullary oncology. Beyond nodal positivity per se, the LNR provides a reproducible, pathology-based metric that incorporates both disease extent and the adequacy of nodal harvest. Using ROC/Youden methods, we identified 0.154 as the optimal cutoff; LNR ≥ 0.154 independently stratified mortality after adjustment for morphology and other covariates, underscoring its additive prognostic value to conventional N staging [14,15,16,17,18,19,20].

Tumor markers and cholestatic indices complemented the clinicopathologic profile. As expected, CA 19-9—a widely used but nonspecific biomarker subject to elevation in cholestasis—varied across groups, with higher levels in PAN; prior work from our group associated elevated preoperative CA 19-9 with an approximately fourfold increase in mortality risk among pancreatic tumors [21,22]. In this cohort, markers of cholestasis were more pronounced in PAN and, to a lesser extent, PB; GGT, in particular, was consistently higher in these two groups when compared with INT, a pattern concordant with its ductal origin and sensitivity to biliary obstruction [23].

From a pathobiologic perspective, the overlap between PB and PAN extended beyond survival to adverse histological features: both exhibited higher frequencies of lymphovascular and perineural invasion and more positive margins, whereas INT displayed a more favorable distribution of these factors. The greater perineural and lymphovascular infiltration in pancreatic primaries likely reflects later symptom onset and the rich sympathetic/parasympathetic innervation of the pancreas, contributing to their aggressive phenotype [6]. These co-segregating features plausibly mediate part of the morphology–survival relationship observed in our study.

Clinically, our results have two direct implications. First, routine reporting of morphological subtype (INT vs. PB) should accompany anatomic site and TNM in surgical pathology, given its independent prognostic value and potential to inform adjuvant therapy decisions. Second, integrating LNR with morphology offers pragmatic risk granularity for postoperative counseling and trial stratification, using data already available in standard workflows. Notably, the similarity between PB and PAN supports considering pancreatic-type adjuvant regimens for ampullary PB tumors, consistent with current pancreatic cancer guidelines [24,25]. In contrast, for INT tumors—which are closer to a colorectal-like biology—gemcitabine-based strategies may be less effective, suggesting that fluoropyrimidine-based regimens should be prioritized when chemotherapy is indicated [26,27,28,29].

Our data should be interpreted in light of several limitations. The retrospective design entails potential selection and information biases. Immunohistochemistry was applied selectively (reserved for equivocal cases after the 33rd case), which could introduce misclassification in borderline tumors. Adjuvant chemotherapy was not analyzed and may confound survival comparisons. Furthermore, we did not evaluate distal bile duct or duodenal primaries due to small numbers. Finally, while comorbidities were highly prevalent in our cohort (≈63–76%), their impact on OS was not significant here, in contrast to a prior study from our institution focusing solely on pancreatic head adenocarcinoma, which found worse survival with older age and comorbidities; differences from published prevalence (≈20–30%) likely reflect referral patterns and population differences [15,21,30]. A larger cohort of pancreatic ductal adenocarcinoma cases and an extended follow-up period would increase the statistical power and provide a more comprehensive understanding of long-term outcomes. However, additional cases with complete immunohistochemical profiling and verified clinical data are not yet available in our institutional database, and long-term recurrence data were not uniformly available for the entire study period. We plan to include more cases in future analyses, and multicenter collaborations are also envisioned to validate and expand upon our findings.

In summary, morphological subtype (INT, PB, PAN) and LNR independently stratify survival after curative-intent PD for periampullary adenocarcinoma. Systematic incorporation of both—alongside TNM—can sharpen postoperative risk assessment, inform adjuvant therapy selection (particularly aligning PB with pancreatic-type regimens), and improve stratification in clinical trials, addressing the persistent heterogeneity observed even within the same anatomic site [14,15,16,17,18,19,20,21,24,25,26,27,28,29]. Indeed, emerging evidence indicates that elucidating the complex crosstalk between cancer cells and the nervous system may pave the way for more effective and targeted therapies. Furthermore, advances in lipid-based nanomaterials have demonstrated remarkable potential in overcoming drug resistance, highlighting their promise for future therapeutic strategies aimed at reversing treatment resistance in these aggressive tumors [31,32,33].

## 5. Conclusions

In this single-center retrospective cohort, morphological subtype and lymph node ratio emerged as independent predictors of overall survival in patients undergoing curative-intent pancreaticoduodenectomy for periampullary adenocarcinomas. The intestinal subtype was associated with more favorable pathological features and significantly longer survival compared to pancreatobiliary and pancreatic types, which exhibited increasingly aggressive characteristics and poorer outcomes.

Integrating morphological classification and lymph node ratio into postoperative prognostic assessment enhances risk stratification beyond conventional TNM staging. These findings support the inclusion of morphological subtype reporting in routine pathology and suggest that pancreatobiliary-type ampullary tumors may benefit from adjuvant strategies similar to those used for pancreatic cancer. Future multicenter studies are needed to validate these results and further refine personalized treatment approaches for periampullary malignancies.

## Figures and Tables

**Table 1:** Distribution of clinical and demographic variables, postoperative complications, and mortality, and comparison among groups according to different histopathological subtypes in patients with periampullary tumors undergoing curative-intent surgical resection.

	Histopathological Phenotype				*p*-Value		
Variables	INT (n = 34)	PB (n = 33)	PAN (n = 53)	*p*-Value	INT vs. PB	INT vs. PAN	PB vs. PAN
**Age (years)**							
Mean ± SD (min.; max.)	64.7 ± 8.8 (46; 78)	61.1 ± 12.4 (30; 84)	59.8 ± 11.1 (34; 89)	NA ^$^	0.169	0.036	0.549
Median: variance (QI, QIII)	67:32 (58, 72)	62:54 (53, 68.5)	60:55 (51, 67)	0.005			
<60	9 (26.5%)	14 (42.4%)	26 (53.1%)	0.117			
≥60	25 (73.5%)	19 (57.6%)	27 (50.9%)				
**Gender**							
Male	19 (55.9%)	12 (36.4%)	33 (62.3%)	0.061	0.109	0.554	0.019
Female	15 (44.1%)	21 (66.6%)	20 (37.7%)				
**Smoking**							
No	18 (52.9%)	28 (69.7%)	24 (45.3%)	0.086	0.159	0.486	0.027
Yes	16 (47.1%)	10 (30.3%)	29 (54.7%)				
**Alcoholism**							
No	26 (76.5%)	28 (84.8%)	30 (56.6%)	0.013	0.386	0.059	0.007
Yes	8 (23.5%)	5 (15.2%)	23 (43.4%)				
**ASA** **^#^**							
I	8 (23.5%)	12 (36.4%)	16 (30.2%)	0.731	0.424	0.596	0.839
II	19 (55.9%)	17 (51.5%)	30 (56.6%)				
III	7 (20.6%)	4 (12.1%)	7 (13.2%)				
**Clavien-Dindo** **^&^**							
No complications	16 (47.1%)	21 (63.6%)	26 (49.1%)	0.703	0.561	0.039	0.041
Grades I + II + IIIA	13 (38.2%)	9 (27.3%)	23 (43.4%)				
Grades IIIB + IV	1 (2.9%)	1 (3.0%)	1 (1.9%)				
Grade V	4 (11.8%)	2 (6.1%)	3 (5.7%)				
**Postoperative death (Within 30 days)**							
No	30 (88.2%)	31 (93.9%)	50 (94.3%)	0.536	0.414	0.425	0.939
Yes	4 (11.8%)	2 (6.1%)	3 (5.7%)				

INT: intestinal-type ampullary adenocarcinoma; PB: pancreatobiliary-type ampullary adenocarcinoma; PAN: pancreatic ductal adenocarcinoma; SD denotes standard deviation; Q: quartile ^$^ Not applicable. ^#^ American Society of Anesthesiologists (ASA) classification. ^&^ According to the Clavien–Dindo classification; n: number; vs.: *versus*.

**Table 2:** Distribution of histopathological variables and comparison among groups according to different histopathological subtypes in patients with periampullary tumors submitted to curative-intent surgical resection.

	Histopathological Phenotype				*p*-Value		
Variables	INT (n = 34)	PB (n = 33)	PAN (n = 53)	*p*-Value	INT vs. PB	INT vs. PAN	PB vs. PAN
**T stage ^†^**							
T1	19 (55.9%)	3 (9.1%)	4 (7.5%)	<0.001	<0.001	<0.001	<0.001
T2	9 (26.5%)	16 (48.5%)	11 (20.8%)				
T3	6 (17.6%)	14 (42.4%)	28 (52.8%)				
T4	0 (0.0%)	0 (0.0%)	10 (18.9%)				
**N stage ^†^**							
N0	26 (76.5%)	18 (54.5%)	16 (30.2%)	0.001	0.127	<0.001	0.080
N1	4 (11.8%)	10 (30.3%)	24 (45.3%)				
N2	4 (11.8%)	5 (15.2%)	13 (24.5%)				
**M stage ^†^**							
M0	34 (100%)	32 (97.0%)	48 (90.6%)	0.119	0.005	<0.001	<0.001
M1	0 (0.0%)	1 (3.0%)	5 (9.4%)				
**TNM staging ^†^**							
0	9 (26.5%)	0 (0.0%)	0 (0.0%)	<0.001	0.005	<0.001	<0.001
I	15 (44.1%)	14 (42.4%)	4 (7.5%)				
II	2 (5.9%)	4 (12.1%)	26 (49.1%)				
III	8 (23.5%)	14 (42.4%)	18 (34.0%)				
IV	0 (0.0%)	1 (3.0%)	5 (9.4%)				
**N positivity**							
Negative	26 (76.5%)	18 (54.5%)	16 (30.2%)	<0.001	0.059	<0.001	0.025
Positive	8 (23.5%)	15 (45.5%)	37 (69.8%)				
**Degree of differentiation**							
Well/Moderate differentiation	34 (100%)	32 (97.0%)	49 (92.5%)	0.223	0.090	0.001	0.043
Poor differentiation	0 (0.0%)	1 (3.0%)	4 (7.5%)				
**Angiolymphatic invasion**							
Absent	30 (88.2%)	12 (36.4%)	8 (15.1%)	<0.001	0.062	<0.001	0.023
Present	4 (11.8%)	21 (63.6%)	45 (84.9%)				
**Perineural invasion**							
Absent	30 (88.2%)	23 (69.7%)	2 (3.8%)	<0.001	0.062	<0.001	<0.001
Present	4 (11.8%)	10 (30.3%)	51 (96.2%)				
**Surgical margins**							
Negative	34 (100%)	32 (97%)	34 (64.2%)	<0.001	0.09	<0.001	0.043
Positive	0 (0.0%)	1 (3.0%)	19 (35.8%)				
**Lymph node ratio**							
<0.154	31 (91.2%)	23 (69.7%)	34 (64.2%)	0.018	0.074	<0.001	0.059
≥0.154	3 (8.8%)	10 (30.3%)	19 (35.8%)				

INT: intestinal-type ampullary adenocarcinoma; PB: pancreatobiliary-type ampullary adenocarcinoma; PAN: pancreatic ductal adenocarcinoma; ^†^ According to the AJCC Classification, 2017, 8th edition.

**Table 3:** Histopathological variables and mean survival of patients with periampullary tumors undergoing curative-intent surgical resection.

Variables	Mean ± SD (Months)	95% Confidence Interval		*p*-Value
		Minimum	Maximum	
**T stage** ^†^				
T1	98.64 ± 11.18	76.76	120.56	<0.001
T2	70.51 ± 9.23	52.48	88.61	
T3	33.34 ± 6.59	20.43	46.25	
T4	30.22 ± 3.80	22.77	37.68	
**N stage** ^†^				
N0	78.68 ± 7.46	64.06	93.30	<0.001
N1	29.20 ± 5.62	18.19	40.23	
N2	26.51 ± 4.46	17.78	35.25	
**N Positivity**				
Negative	78.68 ± 7.46	64.06	93.30	<0.001
Positive	29.42 ± 4.66	20.29	38.54	
**Lymph node ratio**				
<0.154	73.07 ± 6.59	60.15	86.00	<0.001
≥0.154	19.32 ± 2.79	13.85	24.79	
**Angiolymphatic invasion**				
Absent	94.29 ± 7.66	79.28	109.30	<0.001
Present	30.10 ± 4.19	21.89	38.31	
**Perineural invasion**				
Absent	87.93 ± 7.60	73.02	102.83	<0.001
Present	26.84 ± 3.92	19.17	34.52	
**Degree of differentiation**				
Well	81.83 ± 11.72	58.87	104.79	0.013
Moderate	47.94 ± 5.69	36.79	59.08	
Poor	22.60 ± 9.54	3.89	41.30	
**Anatomical location**				
Major duodenal papilla Pancreas	83.79 ± 7.36	69.36	98.21	<0.001
	22.70 ± 5.43	18.31	27.09	
**Morphological classification**				
Intestinal	108.76 ± 8.00	93.00	124.51	<0.001
Pancreatobiliary	62.00 ± 8.90	44.51	79.56	
Pancreas	22.70 ± 2.23	18.31	27.09	

^†^ According to the AJCC Classification, 2017, 8th edition.

**Table 4:** Overall survival means and survival according to morphological classification, with survival rates at 12, 36, and 60 months in patients with periampullary tumors undergoing curative-intent surgical resection.

Groups	Mean ± SD (Months)	12 Months (% Survival)	36 Months (% Survival)	60 Months (% Survival)	*p*-Value
**Intestinal**					
	108.76 ± 8	96.3%	91.2%	76.0%	
**Pancreatobiliary**					
	62.0 ± 8.9	78.6%	58.2%	49.3%	<0.001
**Pancreas**					
	22.7 ± 2.23	81.3%	18.2%	0.0%	
**Overall**					
	56.37 ± 5.43	76.9%	48.8%	37.0%	

SD: Standard Deviation.

**Table 5:** Multivariate analysis and Hazard Ratio (HR) of patients with periampullary tumors undergoing curative-intent surgical resection.

Variables	HR	95% Confidence Interval	*p*-Value
**Lymph node ratio**			
<0.154	1.00	-	-
≥0.154	1.93	1.11–3.35	0.018
**Morphological Classification**			
Intestinal subtype	1.00	-	-
Pancreatobiliary subtype	4.41	1.25–15.53	0.021
Pancreatic subtype	13.96	3.99–48.75	<0.001

## Data Availability

The original contributions presented in this study are included in the article. Further inquiries can be directed to the corresponding author.

## Abbreviations

The following abbreviations are used in this manuscript:

AJCC	American Joint Committee on Cancer
ALT	Alanine aminotransferase
AST	Aspartate aminotransferase
ASA	American Society of Anesthesiologists
CA 19-9	Carbohydrate antigen 19-9
CI	Confidence interval
GGT	Gamma-glutamyl transferase
H&E	Hematoxylin and eosin
HR	Hazard ratio
INT	Intestinal subtype
LNR	Lymph node ratio
OS	Overall survival
PB	Pancreatobiliary subtype
PAN	Pancreatic ductal adenocarcinoma
PNI	Perineural invasion
ROC	Receiver operating characteristic
R0/R1	Margin status (negative/positive)
SD	Standard deviation
UFMG	Federal University of Minas Gerais
